# Inguinal Herniation of a Transplant Kidney Ureter: A Case Report

**DOI:** 10.5812/iranjradiol.10251

**Published:** 2012-12-27

**Authors:** Marina Pourafkari, Mishka Ghofrani, Majid Riahi

**Affiliations:** 1 Department of Radiology, Faculty of Medicine, Shahid Beheshti University of Medical Sciences, Tehran, Iran; 2 Shahid Beheshti University of Medical Sciences, Tehran, Iran

**Keywords:** Ureteral Obstruction, Kidney, Hernia, Inguinal

## Abstract

Ureteral obstruction is relatively common after renal transplantation. A rare cause is the inguinal herniation of the transplant ureter. We report a case of late allograft renal transplant failure due to ureteral herniation as well as ureterovesical junction stenosis.

##  1. Background

Ureteral obstruction is a relatively common complication of renal transplantation and it comprises almost half of postoperative urologic complications. Most complications occur at the ureterovesical junction and are secondary to technical causes and ureteric ischemia (1, 2). A rare cause of obstructive uropathy is herniation of the transplant ureter into an inguinal hernia (2-4) and it should be considered in the differential diagnoses of an obstructed transplant kidney. We present a rare case of late ureteral obstruction in a transplant kidney due to stenosis at ureterovesical junction as well as inguinal herniation of the transplant ureter.

## 2. Case Presentation

**Figure 1:**
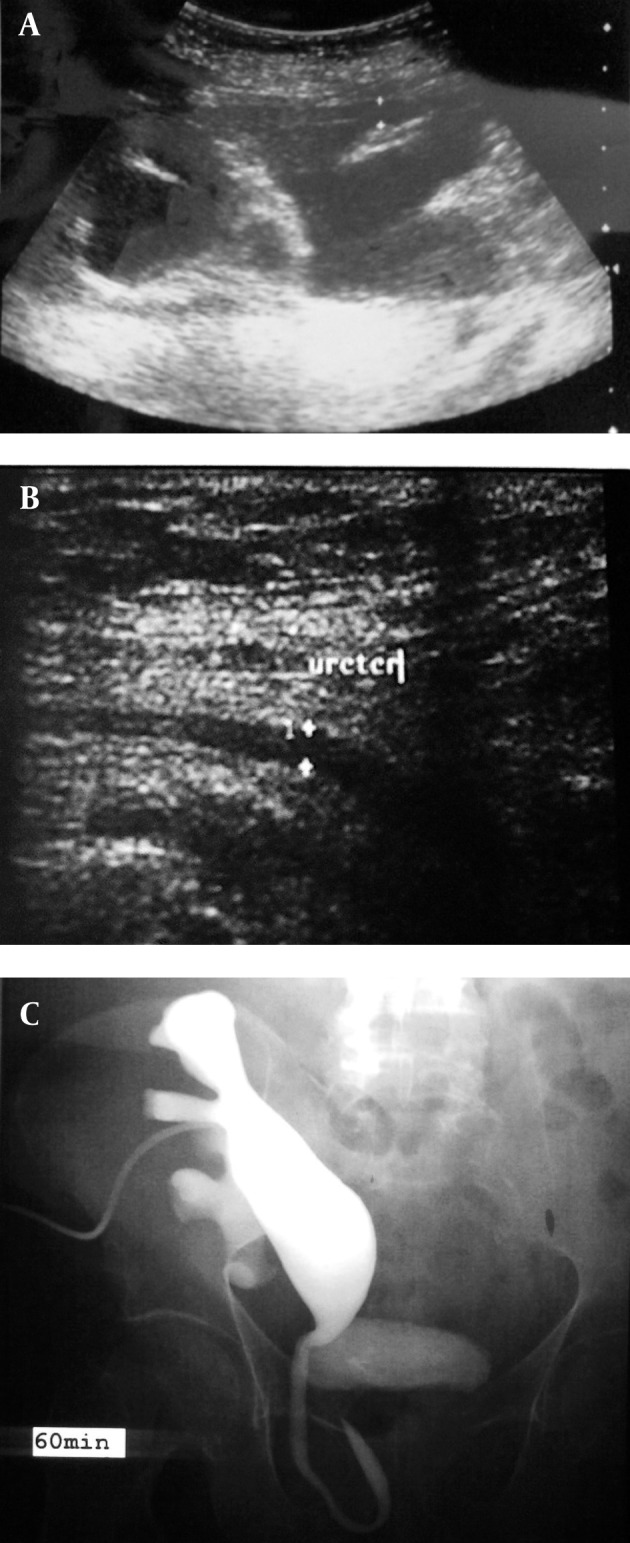
A 50-year-old man with allograft kidney transplant presenting with renal failure. A, Ultrasonography shows severe hydronephrosis of the transplant kidney. B, Dilated ureter in the inguinal canal is evident on ultrasonography. C, Nephrostogram shows the dilated pyelocaliceal system and a ureteral loop in the inguinal canal. The ureterovesical junction is not well visualized.

A 50 year old man with a history of diabetes, hyperlipidemia, cardiac arrhythmia, BCC and SCC of lids and allograft kidney transplantation 12 years previously presented with weakness, drowsiness and decreased urinary force and caliber. Serum creatinine was increased from 1.8mg/dL postoperatively to 2.6 mg/dL. Initial ultrasonography revealed severe hydronephrosis of the transplant kidney and ureteral dilation. The dilated ureter could be followed down to the scrotum with abundant surrounding echogenic fat (Figure 1A & B). Radionuclide scanning also showed severe hydronephrosis of the transplant kidney, moderate to severe function impairment and evidence of severe obstruction most probably at ureterovesical junction. A percutaneous nephrostomy was performed. The nephrostogram demonstrated the herniated ureter in the right inguinal canal, stenosis at ureterovesical junction and poor bladder filling (Figure 1C).

Ureterovesical junction stenosis was dilated by flexible antegrade ureteroscopy and a double-J catheter was placed. Serum creatinine decreased to 1.4mg/dL and on follow up sonography hydronephrosis was relieved. Surgery was not possible due to the medical condition. The patient died about four months later due to cardiac arrest.

## 3. Discussion

The dilated ureter in the hernial sac of the patient suspected us of ureteral herniation as a possible cause of obstruction evidenced by the severe hydronephrosis, more details were obtained by the nephrostogarphy. Antegrade uretroscopy was done to alleviate the ureterovesical junction stenosis and double-J catheter insertion.

Ureteral obstruction and urinary fistulae account for 95% of urologic complications in renal transplantation (3). Herniation of the transplant ureter is a rare complication and can cause or exacerbate ureteral obstruction (2, 4). Ureteroingunal hernias of native kidneys are classified into two major groups, depending on the presence or absence of a concomitant peritoneal hernia sac. The para (intra) peritoneal type is accompanied by a herniated peritoneal sac adjacent to the ureter. In the rare extraperitoneal type, only the ureter hernaites through the inguinal canal, frequently surrounded by abundant retroperitoneal tissue (2, 5, 6).

Ultrasound is usually the first examination to diagnose obstructive uropathy in patients with renal implantation. If physical examination demonstrates an inguinal hernia on the same side as an obstructed transplanted kidney, sonographic evaluation should be performed to determine whether the ureter is obstructed at this level (2). In most cases of described ureteroinguinal hernia the diagnosis was made by IVP (5-12). It reveals superimposition of the afferent and efferent limbs of the ureter as it traverses the hernia sac, producing the pathognomonic “loop -the-loop” or “curlicue” ureter. CT urography, an important alternative to IVP, can demonstrate the uretroinguinal hernia and accompanying pathologic changes rapidly and with better resolution (5).

Factors that may contribute to inguinal hernaition of the transplant kidney are the existence of a redundant ureter (as in our patient), placement of donor ureter over the spermatic cord and obesity (1-3). Although surgical repair is recommended in all cases of ureteroinguinal hernia, conservative treatment can be a satisfactory alternative in patients with high risk of surgery (8). As is the case with our patient, renal function returned to normal after conservative management by ureteroscopic dilation and catheter insertion, yet no long-term follow-up was possible as the patient died four months later.
